# Complex regional pain syndrome: The matter of white matter?

**DOI:** 10.1002/brb3.647

**Published:** 2017-04-05

**Authors:** Jaakko Hotta, Guangyu Zhou, Hanna Harno, Nina Forss, Riitta Hari

**Affiliations:** ^1^ Department of Neuroscience and Biomedical EngineeringAalto UniversityEspooFinland; ^2^ Aalto NeuroImagingAalto UniversityEspooFinland; ^3^ Clinical Neurosciences, NeurologyUniversity of Helsinki and Department of Neurology, Helsinki University HospitalHelsinkiFinland; ^4^ Department of NeurologyNorthwestern UniversityChicagoILUSA; ^5^ Pain ClinicDepartment of Anesthesiology, Intensive Care and Pain MedicineUniversity of Helsinki and Helsinki University HospitalHelsinkiFinland; ^6^ Department of ArtAalto UniversityHelsinkiFinland

**Keywords:** central nervous system, chronic pain, connectivity, diffusion tensor imaging, motor skills

## Abstract

**Introduction:**

Many central pathophysiological aspects of complex regional pain syndrome (CRPS) are still unknown. Although brain‐imaging studies are increasingly supporting the contribution of the central nervous system to the generation and maintenance of the CRPS pain, the brain's white‐matter alterations are seldom investigated.

**Methods:**

In this study, we used diffusion tensor imaging to explore white‐matter changes in twelve CRPS‐type‐1 female patients suffering from chronic right upper‐limb pain compared with twelve healthy control subjects.

**Results:**

Tract‐based spatial‐statistics analysis revealed significantly higher mean diffusivity, axial diffusivity, and radial diffusivity in the CRPS patients, suggesting that the structural connectivity is altered in CRPS. All these measures were altered in the genu, body, and splenium of corpus callosum, as well as in the left anterior and posterior and the right superior parts of the corona radiata. Axial diffusivity was significantly correlated with clinical motor symptoms at whole‐brain level, supporting the physiological significance of the observed white‐matter abnormalities.

**Conclusions:**

Altogether, our findings further corroborate the involvement of the central nervous system in CRPS.

## 1 Introduction

Although the complete pathophysiology of complex regional pain syndrome (CRPS) is still poorly understood, previous brain‐imaging studies have suggested the involvement of central nervous system in the maintenance and progression of the pain (Marinus et al., 2011). For example, the CRPS patients can have altered hand representation as a sign of cortical reorganization (Di Pietro, Stanton, Moseley, Lotze, & McAuley, 2015; Juottonen et al., 2002), and some of the observed brain alterations are associated with clinical characteristics of CRPS, such as motor dysfunction (Maihöfner et al., 2007).

More recently, morphometric changes have been found in the CRPS patients' brains with anatomical T1 MRI (Baliki, Schnitzer, Bauer, & Apkarian, 2011; Barad, Ueno, Younger, Chatterjee, & Mackey, 2014; Geha et al., 2008; Pleger et al., 2014), and we have observed the enlargement of choroid plexus (Zhou, Hotta, Lehtinen, Forss, & Hari, 2015), in line with the suggestion that neuroinflammation might play a role in the pathophysiology of CRPS (Linnman, Becerra, & Borsook, 2013). In neuroinflammatory diseases, brain's white matter can possess microstructural abnormalities although it appears normal in anatomical T1 MRI (Filippi et al., 2012).

Diffusion tensor imaging (DTI) has revealed plastic changes in the brain's white matter after motor training in healthy subjects (Bengtsson et al., 2005; Scholz, Klein, Behrens, & Johansen‐Berg, 2009; Valkanova, Eguia Rodriguez, & Ebmeier, 2014) as well as in patients with traumatic brain injury (Drijkoningen et al., 2015). DTI can also detect effects of neuroinflammation (Pasternak, Kubicki, & Shenton, 2015; Quarantelli, 2015). Thus, considering the motor dysfunction, maladaptive neuroplasticity, and neuroinflammation in CRPS (Marinus et al., 2011), DTI has the potency to detect CRPS‐related primary and secondary white‐matter changes.

Two studies have previously analyzed DTI in CRPS, both focusing on fractional anisotropy (FA). In whole‐brain analysis, van Velzen, Rombouts, van Buchem, Marinus, and van Hilten (2016) found no FA abnormalities, whereas Geha et al. (2008) showed FA to be lowered in the left cingulum; the subsequent region‐of‐interest analysis revealed decreased axial diffusivity (AD) and increased radial diffusivity (RD) in the same area. However, because FA is sometimes insensitive to changes in other DTI measures (Acosta‐Cabronero, Williams, Pengas, & Nestor, 2010; Hasan, 2006), it would be important to examine each of these measures in a whole‐brain manner.

In the present study, we performed whole‐brain tract‐based spatial‐statistics (TBSS) analysis of FA, mean diffusivity (MD), AD, and RD to further examine changes in white‐matter integrity in CRPS type‐1 patients. To address the functional relevance of these changes, we also explored the associations between the motor dysfunction and disease duration, and the DTI measures.

## 2 Materials and Methods

### 2.1 Participants

Twelve female patients with chronic type‐1 CRPS affecting right‐dominant upper‐limb (mean ± *SD* age 46 ± 6 years, median 46, range 35–57) participated in the study. As the control group, we selected twelve optimally age‐ and scanner‐matched (see Image acquisition) subjects (44 ± 10 years, median 45, range 25–60) from sixteen successfully scanned healthy female participants. The groups did not differ in age (*p* > .05, Wilcoxon rank‐sum test). All subjects were right‐handed by the Edinburg Handedness Inventory and self‐report. Before participation, all subjects gave informed written consent in accordance with the Declaration of Helsinki. The study protocol was approved by the Ethics Committee of the Helsinki and Uusimaa Hospital District.

Eight patients were recruited from the Pain Clinic at Helsinki University Hospital where we searched the patient records from year 2007 to 2013 for 18–65 years old patients with chronic upper‐limb CRPS (disease duration > 6 months). Four patients were recruited from other clinics treating CRPS in the Uusimaa district. After interviewing all suitable candidates, we clinically examined those eligible for fulfilling both the current diagnostic criteria for CRPS (Harden, Bruehl, Stanton‐Hicks, & Wilson, 2007) and our criteria stipulating (1) severe pain (pain intensity greater than four on 11‐point numeric scale); (2) right‐handedness; (3) no other major psychiatric or neurological diseases, or drug/alcohol addiction; (4) no contraindication for MRI.

In clinical examination, the diagnostic symptoms and signs were evaluated by an experienced neurologist (author H.H.) with a subspecialty in pain medicine. The disease duration was a mean ± *SD* of 5.8 ± 4.5 years. Table 1 summarizes details of clinical examination and demographic data.

**Table 1:**
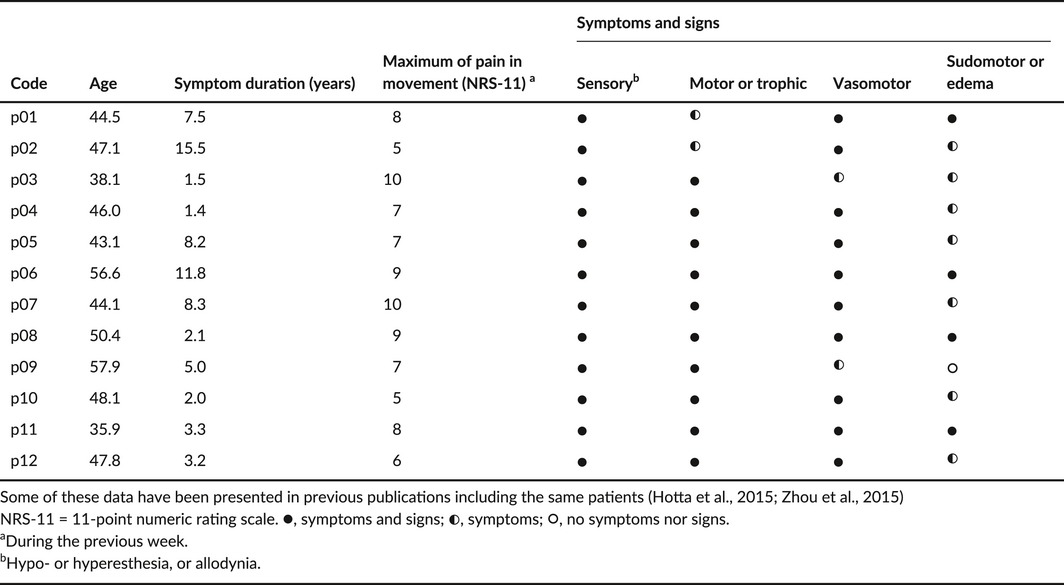
Demographic and clinical examination data of complex regional pain syndrome patients

### 2.2 Motor‐symptom‐severity index

To evaluate the overall severity of motor‐symptoms in the affected hand, we created motor‐symptom‐severity index (MSSI) as a compound score of five subjective and objective measures: (1) movement‐related pain (11‐point numeric rating scale) and (2) upper‐limb disability (Disabilities of Arm, Shoulder, and Hand questionnaire i.e., DASH, Institute for Work & Health [Hudak, Amadio, & Bombardier, 1996]) questionnaires, and (3) hand dexterity (nine‐hole peg test), (4) total active range of wrist movement, and (5) grip strength assessed by a skilled physiotherapist. We calculated MSSI as the average *z*‐score of these five components after the grip strength and wrist AROM z‐scores had been multiplied by −1, so that for all components a higher score implied more severe motor symptoms. See Supporting Information for details.

### 2.3 Image acquisition

Diffusion tensor imaging was performed at the Advanced Magnetic Imaging Centre of Aalto NeuroImaging, Aalto University. Because of the scanner upgrade in‐between our data collection, the first eight patients and eight healthy control subjects were measured with a 16‐channel head coil GE HDxt 3T scanner (GE Healthcare, Milwaukee, WI, USA) and the remaining four patients and four healthy control subjects with a 30‐channel head coil Magnetom Skyra 3T scanner (Siemens Healthcare, Erlangen, Germany). We acquired the diffusion‐weighted images in 60/64 (GE/Siemens) noncollinear diffusion‐sensitizing‐gradient directions (*b* = 1,000 s/mm^2^) together with 4/1 (GE/Siemens) non‐diffusion‐weighted images (*b* = 0 s/mm^2^) in 51 axial slices (no gaps) with voxel size of 1.875 × 1.875 × 3.0 mm^3^.

### 2.4 Tract‐based spatial statistics

The preprocessing of DTI data included motion and eddy‐current correction, and brain extraction using FSL toolkit (http://www.fmrib.ox.ac.uk/fsl/; Smith et al., 2004). The B‐matrices were also rotated (Leemans & Jones, 2009). A brain mask was created from the diffusion‐weighted image with b‐value of zero, using the FSL's brain extraction tool (Smith, 2002).

Tensors were estimated on the corrected data within the brain mask using FSL's *dtifit* and the resulting images were converted into DTI‐TK (http://www.nitrc.org/projects/dtitk) format for tensor‐based spatial normalization (Zhang et al., 2007). First, population‐specific tensor template was bootstrapped using the IXI aging DTI template (Zhang, Yushkevich, Rueckert, & Gee, 2010). Individual tensor image was then registered to the population‐specific template that was mapped to the IIT (Illinois Institute of Technology) DTI human template (v3; Zhang, Peng, Dawe, & Arfanakis, 2011) with rigid, affine and finally diffeomorphic registrations. Finally, aligned individual tensor images were wrapped to the population‐specific template in standard space. This tensor‐based normalization has been shown to be superior in detecting white‐matter differences compared with low‐dimensional registration using scalar values, such as FA (Wang et al., 2011; Zhang et al., 2007).

The FA, MD, AD, and RD maps were reconstructed from the spatially normalized tensor images for each participant. These DTI parameters were compared voxel‐by‐voxel with TBSS (Smith et al., 2006), as part of FSL. Briefly, the mean FA skeleton image that represents the center of tracts, consistent across subjects, was obtained by thinning the across‐subjects averaged FA image (threshold of 0.2). Then, each subject's aligned FA, MD, AD, and RD images were projected onto the mean FA skeleton.

Between‐group comparisons of the resulting skeletonized data were conducted using permutation test (FSL's Randomise v2.1; 10,000 permutations), with multiple comparisons corrected using threshold‐free cluster enhancement method (Smith & Nichols, 2009). We considered a difference to be statistically significant at a corrected *p* < .05. Confounding factors of age and scanner were included as covariates. Statistically significant tracts were labeled according to the white‐matter atlas from Johns Hopkins University (JHU ICBM‐DTI‐81 white‐matter labels).

Whole‐brain skeletal FA, MD, AD, and RD were calculated by averaging over the FA skeleton. Between‐group comparisons in these values were performed using unpaired two‐tailed two‐sample *t* tests. Age and scanner were regressed out using multiple linear regression before the comparison analysis.

To evaluate the bias caused by head motion, the motion‐correction parameters (absolute displacement and mean absolute translations) were also compared between the two groups using unpaired two‐tailed two‐sample *t* test. The mean translation values were calculated across all three axes.

### 2.5 Correlation between DTI parameters and clinical characteristics

Pearson coefficients were computed between the whole‐brain skeletal DTI and clinical characteristics including the disease duration and MSSI (Matlab, Mathworks Inc.). Voxelwise correlation analyzes between DTI measures and clinical characteristics were performed using FSL's Randomise (v2.1, 10,000 permutations) and multiple correlations were corrected using the treshold‐free cluster enhancement method. Age and scanner were included as covariates.

## 3 Results

### 3.1 DTI changes

**Figure 1:**
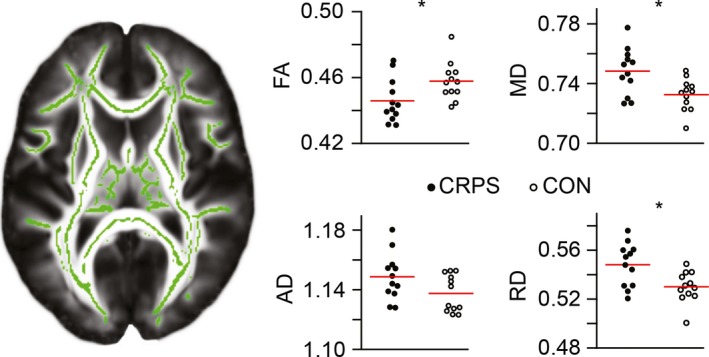
Whole‐brain diffusion tensor imaging (DTI) skeleton (left, shown in green) and mean skeletal DTI parameters for fractional anisotropy (FA), mean diffusivity (MD), axial diffusivity (AD), and radial diffusivity (RD) in the complex regional pain syndrome patients (CRPS) and healthy control subjects (CON). Each symbol refers to one subject. The asterisks refer to statistically significant differences between the groups. Data were adjusted for age and scanner

Figure 1 (left) shows the DTI skeleton at one axial plane and (right) the subject‐wise whole‐brain skeletal parameters (mean FA, MD, AD, and RD) for all the CRPS patients and healthy control subjects. The patients had lower mean FA (Cohen's *d* = 0.97, *p* = .027, two‐tailed two‐sample *t* test), and higher mean MD (*d* = 1.19, *p* = .008) and RD (*d* = 1.18, *p* = .009) than the control subjects. The groups did not differ in mean AD (*d* = 0.79, *p* = .066). The mean AD showed a statistically significant positive correlation with MSSI in the patients (*r* = .67, *p* = .018). No other significant correlations were present between the DTI parameters and MSSI or disease duration.

**Figure 2:**
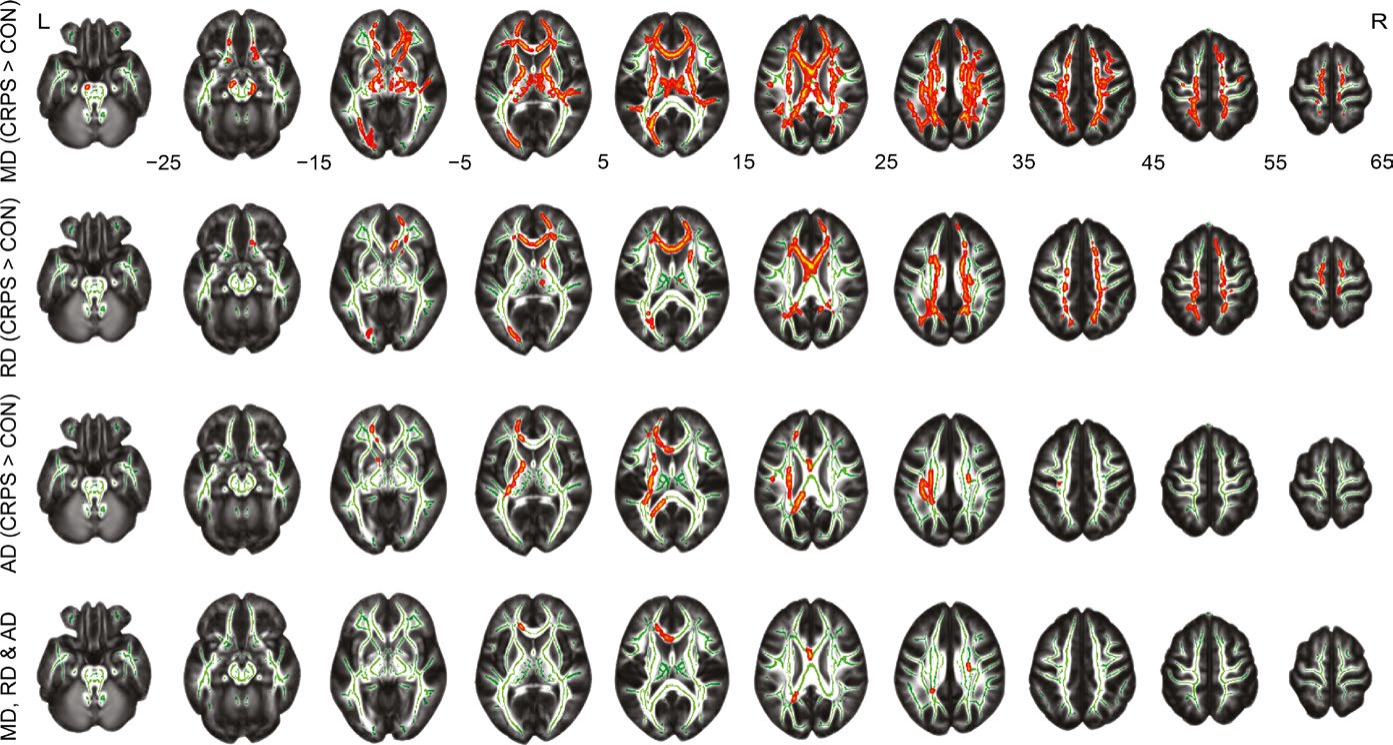
Results of tract‐based spatial‐statistics analysis. The red–yellow colors show voxels where complex regional pain syndrome (CRPS) patients have increased mean diffusivity (MD; top row), increased radial diffusivity (RD; 2nd row) or increased axial diffusivity (AD; 3rd row) compared with healthy control subjects (CON) (*p* < .05, threshold‐free cluster enhancement corrected). The bottom row shows the overlap of differences. All voxels with group‐differences were enlarged for better visualization using tbss_fill. The study‐specific mean fractional anisotropy and the corresponding white‐matter skeleton (green) were used as background images. R, right hemisphere; L, left hemisphere

Figure 2 illustrates the TBSS findings and Table 2 summarizes the results. MD (top row) was higher in the CRPS patients than control subjects in widespread regions of the white‐matter skeleton (23% of all tested voxels, *p* < .05, corrected with treshold‐free cluster enhancement). Similarly, RD was increased in patients in multiple regions (second row from top; 10% of all tested voxels), whereas AD increases appeared more locally, mainly in the left hemisphere (third row from top; 3% of all tested voxels). The brain regions showing statistically significant between‐group differences for all MD, RD, and AD (lowest row) included the corpus callosum (genu, body, and splenium) and the corona radiata (left anterior, right superior, and left posterior). The groups did not differ in local skeletal FA. For further statistics, see Table 2.

**Table 2:** Tract‐based spatial‐statistics analysis results

Region	MD (CRPS > CONTROL, No. clusters = 1)			AD (CRPS > CONTROL, No. clusters = 8)			RD (CRPS > CONTROL, No. clusters = 9)		
	No. voxels	Peak coordinate (*x, y, z)*	corr. *p*	No. voxels	Peak coordinate (*x, y, z*)	corr. *p*	No. voxels	Peak coordinate (*x, y, z*)	Corr. *p*
Corpus callosum									
Genu	1,140	−6, 24, 12	.014	144	−14, 33, 7	.041	1,127	−4, 26, 8	.024
Body	1,464	−2, 19, 16	.015	93	−5, 18, 17	.045	1,389	13, 3, 31	.025
Splenium	428	19, −33, 33	.015	391	−21, −53, 22	.032	62	20, −40, 30	.031
Internal capsule									
Anterior limb									
R	501	24, ‐6, 18	.019				175	22, 15, 12	.047
L	362	−17, 14, 0	.020	229	−21, −2, 15	.039			
Posterior limb									
R	463	26, −26, 16	.015						
L	449	−22, −12, 8	.021	307	−27, −24, 18	.031			
Retrolenticular part									
R	316	26, −26, 15	.017						
L	67	−27, −28, 10	.023	262	−27, −26, 15	.034			
Superior fronto‐occipital fasciculus^a^									
R	47	21, −9, 19	.019				33	22, 6, 19	.049
Corona radiata									
Anterior									
R	858	17, 35, 10	.016				470	11, 28, −12	.027
L	568	−15, 35, 5	.014	126	−15, 35, 5	.041	310	−15, 35, 5	.025
Superior									
R	903	23, −13, 34	.015	66	23, −12, 36	.040	370	17, −5, 39	.024
L	704	−25, −20, 33	.016	376	−28, −23, 21	.030	258	−17, −3, 37	.026
Posterior									
R	378	22, −31, 38	.015	13	26, −23, 22	.047	179	20, −30, 36	.025
L	307	−22, −35, 38	.016	218	−28, −23, 22	.030	130	−21, −39, 37	.038
Superior longitudinal fasciculus									
R	532	28, −23, 39	.015						
L	437	−35, −24, 36	.016	271	−32, −22, 38	.040			
Fornix^b^	50	2, −15, 16	.020						
External capsule									
R	32	32, −22, 1	.020						
L	23	−28, −16, 18	.023	28	−28, −16, 18	.034			
Posterior thalamic radiation^c^									
L	96	−28, −57, 18	.020	39	−31, −39, 13	.036	29	−28, −57, 18	.039
Cingulum (cingulate gyrus)									
L	14	−8, −21, 32	.037						
Corticospinal tract									
L	62	−8, −20, −31	.030						
Cerebral peduncle									
R	163	15, −22, −5	.020						
L	153	−18, −20, −10	.023						

The peak coordinates are given in MNI space. Only tracts with a number of voxels > 10 are shown. CRPS, complex regional pain syndrome patients; CONTROL, healthy control subjects; MD, mean diffusivity; AD, axial diffusivity; RD, radial diffusivity; corr., corrected; L, left hemisphere; R, right hemisphere.

a Could be a part of the anterior internal capsule.

b The column and body of fornix.

c Includes the optic radiation.

The groups did not differ in head motion during the DT imaging (absolute displacements: CRPS 1.54 ± 0.38 mm; CON 1.53 ± 0.23; *d* = 0.04, *p* = .93; mean absolute translations: CRPS 0.44 ± 0.13 mm; CON 0.40 ± 0.11; *d* = 0.36, *p* = .38).

## 4 Discussion

We examined the white‐matter integrity in the CRPS patients suffering from right upper‐limb pain. The CRPS patients exhibited abnormally high MD values in large parts of white‐matter tracts, coupled with more local increases in RD and AD, and an overall decrease in FA. Although these findings do not specify the underlying microstructural abnormalities, they further support the involvement of the central nervous system in the pathophysiology of CRPS, implying a large‐scale pathology in the structural connectivity of the brains of chronic CRPS patients. Furthermore, the correlations between the severity of motor symptoms and the AD values support the functional significance of the observed white‐matter alterations.

### 4.1 Potential mechanism underlying DTI changes in CRPS

Diffusion tensor imaging abnormalities have been found in a variety of chronic‐pain conditions, such as migraine (Szabó et al., 2012; Yu et al., 2013), temporomandibular disorder (Moayedi et al., 2012), and irritable bowel syndrome (Chen, Blankstein, Diamant, & Davis, 2011). Our current findings indicate that the white‐matter integrity in the chronic CRPS is widely affected. However, the interpretations of the altered DTI parameters are not straightforward because the underlying cellular‐level pathophysiology is incompletely understood (for a review, see e.g., Concha, 2014). For example, increased AD combined with increased RD has been found in several neurological diseases such as multiple sclerosis (Roosendaal et al., 2009), Alzheimer's disease (Acosta‐Cabronero et al., 2010), and amyotrophic lateral sclerosis (Metwalli et al., 2010) with otherwise separate and distinctive pathophysiology.

In the current study, MD was higher in more than one‐fifth of all tested voxels in the CRPS patients compared with healthy subjects. MD changes can reflect e.g., alteration of cellularity, or necrosis (Alexander et al., 2011; Concha, 2014), but MD also increases in vasogenic edema, which could be indicative of e.g., neuroinflammation (Pasternak et al., 2015) that is suspected to occur in CRPS. However, more research is needed to study the role of central inflammaltion in CRPS and its relationship to DTI abnormalities. For example, neuroinflammation‐specific changes of extracellular volume are more likely to be identified with free‐water imaging which is sensitive to water diffusion in the extracellular space (Pasternak et al., 2012, 2015).

The DTI changes can also be experience‐related one (Scholz et al., 2009). Thus, the profound abnormalities in the CRPS patients' daily lives—such as continuous pain, altered sensory input (Rommel, Malin, Zenz, & Jänig, 2001) and reduced motor activity (Schilder et al., 2012)—could explain the large‐scale pathology of the DTI alterations. The positive correlation between motor disability (MSSI) and whole‐brain skeletal AD in the patients would be in line with this.

### 4.2 Caveats

Compared with FA‐intensity‐based registrations, the tensor‐based DTI‐TK registration technique, as used in the present study (see Section 2), improves the alignment of different brains and thereby the detection of group differences (Van Hecke et al., 2007; Wang et al., 2011; Zhang et al., 2007). The differences of these registration methods can affect the detection of group differences with TBSS e.g., in the cingulum bundle (Bach et al., 2014). Despite the present study's methodological advantages and the highly homogeneous patient group (dominant upper‐limb CRPS), we acknowledge that the sample size is relatively small. As DTI studies on the effects of dominant‐arm inactivity or chronic pain from other etiologies are not yet available, the specificity of our findings to CRPS remains to be shown. Future studies with larger number of patients are necessary to understand the spread and significance of white‐matter microstructural abnormalities in the chronic CRPS patients.

## 5 Conclusions

Several DTI parameters were altered in our chronic CRPS patients, supporting the involvement of the central nervous system in the generation and maintenance of the CRPS symptoms. The present results demonstrate specifically the presence of white‐matter alterations in CRPS. The role of white‐matter changes in the clinical course of CRPS should be assessed in future studies.

## Conflict of Interest

No conflict of interest to declare.

## References

[brb3647-bib-0001] Acosta‐Cabronero, J. , Williams, G. B. , Pengas, G. , & Nestor, P. J. (2010). Absolute diffusivities define the landscape of white matter degeneration in Alzheimer's disease. Brain, 133, 529–539. 19914928 10.1093/brain/awp257

[brb3647-bib-0002] Alexander, A. L. , Hurley, S. A. , Samsonov, A. A. , Adluru, N. , Hosseinbor, A. P. , Mossahebi, P. , … Field, A. S. (2011). Characterization of cerebral white matter properties using quantitative magnetic resonance imaging stains. Brain Connectivity, 1, 423–446. 22432902 10.1089/brain.2011.0071 PMC3360545

[brb3647-bib-0003] Bach, M. , Laun, F. B. , Leemans, A. , Tax, C. M. W. , Biessels, G. J. , Stieltjes, B. , & Maier‐Hein, K. H. (2014). Methodological considerations on tract‐based spatial statistics (TBSS). NeuroImage, 100, 358–369. 24945661 10.1016/j.neuroimage.2014.06.021

[brb3647-bib-0004] Baliki, M. N. , Schnitzer, T. J. , Bauer, W. R. , & Apkarian, A. V. (2011). Brain morphological signatures for chronic pain. PLoS One, 6, e26010. 22022493 10.1371/journal.pone.0026010 PMC3192794

[brb3647-bib-0005] Barad, M. J. , Ueno, T. , Younger, J. , Chatterjee, N. , & Mackey, S. (2014). Complex regional pain syndrome is associated with structural abnormalities in pain‐related regions of the human brain. Journal of Pain, 15, 197–203. 24212070 10.1016/j.jpain.2013.10.011 PMC4784981

[brb3647-bib-0006] Bengtsson, S. L. , Nagy, Z. , Skare, S. , Forsman, L. , Forssberg, H. , & Ullén, F. (2005). Extensive piano practicing has regionally specific effects on white matter development. Nature Neuroscience, 8, 1148–1150. 16116456 10.1038/nn1516

[brb3647-bib-0007] Chen, J. Y.‐W. , Blankstein, U. , Diamant, N. E. , & Davis, K. D. (2011). White matter abnormalities in irritable bowel syndrome and relation to individual factors. Brain Research, 1392, 121–131. 21466788 10.1016/j.brainres.2011.03.069

[brb3647-bib-0008] Concha, L. (2014). A macroscopic view of microstructure: Using diffusion‐weighted images to infer damage, repair, and plasticity of white matter. Neuroscience, 276, 14–28. 24051366 10.1016/j.neuroscience.2013.09.004

[brb3647-bib-0009] Di Pietro, F. , Stanton, T. R. , Moseley, G. L. , Lotze, M. , & McAuley, J. H. (2015). Interhemispheric somatosensory differences in chronic pain reflect abnormality of the healthy side. Human Brain Mapping, 36, 508–518. 25256887 10.1002/hbm.22643 PMC6869612

[brb3647-bib-0010] Drijkoningen, D. , Caeyenberghs, K. , Leunissen, I. , Vander Linden, C. , Leemans, A. , Sunaert, S. , … Swinnen, S. P. (2015). Training‐induced improvements in postural control are accompanied by alterations in cerebellar white matter in brain injured patients. NeuroImage Clinical, 7, 240–251. 25610786 10.1016/j.nicl.2014.12.006 PMC4300016

[brb3647-bib-0011] Filippi, M. , Rocca, M. A. , Barkhof, F. , Brück, W. , Chen, J. T. , Comi, G. , … Lassmann, H. (2012). Association between pathological and MRI findings in multiple sclerosis. The Lancet Neurology, 11, 349–360. 22441196 10.1016/S1474-4422(12)70003-0

[brb3647-bib-0012] Geha, P. Y. , Baliki, M. N. , Harden, R. N. , Bauer, W. R. , Parrish, T. B. , & Apkarian, A. V. (2008). The brain in chronic CRPS pain: Abnormal gray‐white matter interactions in emotional and autonomic regions. Neuron, 60, 570–581. 19038215 10.1016/j.neuron.2008.08.022 PMC2637446

[brb3647-bib-0013] Harden, R. N. , Bruehl, S. , Stanton‐Hicks, M. , & Wilson, P. R. (2007). Proposed new diagnostic criteria for complex regional pain syndrome. Pain Medicine, 8, 326–331. 17610454 10.1111/j.1526-4637.2006.00169.x

[brb3647-bib-0014] Hasan, K. M. (2006). Diffusion tensor eigenvalues or both mean diffusivity and fractional anisotropy are required in quantitative clinical diffusion tensor MR reports: Fractional anisotropy alone is not sufficient. Radiology, 239, 611–612; author reply 612–613. 16641362 10.1148/radiol.2392051172

[brb3647-bib-0200] Hotta J. , Harno, H. , Nummenmaa, L. , Kalso, E. , Hari, R. , Forss, N. (2015). Patients with complex regional pain syndrome overestimate applied force in observed hand actions. European Journal of Pain, 19, 1372–1381. 25731614 10.1002/ejp.669

[brb3647-bib-0015] Hudak, P. L. , Amadio, P. C. , & Bombardier, C. (1996). Development of an upper extremity outcome measure: The DASH (disabilities of the arm, shoulder and hand) [corrected]. The Upper Extremity Collaborative Group (UECG). American Journal of Industrial Medicine, 29, 602–608. 8773720 10.1002/(SICI)1097-0274(199606)29:6<602::AID-AJIM4>3.0.CO;2-L

[brb3647-bib-0016] Juottonen, K. , Gockel, M. , Silén, T. , Hurri, H. , Hari, R. , & Forss, N. (2002). Altered central sensorimotor processing in patients with complex regional pain syndrome. Pain, 98, 315–323. 12127033 10.1016/S0304-3959(02)00119-7

[brb3647-bib-0017] Leemans, A. , & Jones, D. K. (2009). The B‐matrix must be rotated when correcting for subject motion in DTI data. Magnetic Resonance in Medicine, 61, 1336–1349. 19319973 10.1002/mrm.21890

[brb3647-bib-0018] Linnman, C. , Becerra, L. , & Borsook, D. (2013). Inflaming the brain: CRPS a model disease to understand neuroimmune interactions in chronic pain. Journal of Neuroimmune Pharmacology, 8, 547–563. 23188523 10.1007/s11481-012-9422-8 PMC3596443

[brb3647-bib-0019] Maihöfner, C. , Baron, R. , DeCol, R. , Binder, A. , Birklein, F. , Deuschl, G. , … Schattschneider, J. (2007). The motor system shows adaptive changes in complex regional pain syndrome. Brain, 130, 2671–2687. 17575278 10.1093/brain/awm131

[brb3647-bib-0020] Marinus, J. , Moseley, G. L. , Birklein, F. , Baron, R. , Maihöfner, C. , Kingery, W. S. , & van Hilten, J. J. (2011). Clinical features and pathophysiology of complex regional pain syndrome. The Lancet Neurology, 10, 637–648. 21683929 10.1016/S1474-4422(11)70106-5 PMC5511749

[brb3647-bib-0021] Metwalli, N. S. , Benatar, M. , Nair, G. , Usher, S. , Hu, X. , & Carew, J. D. (2010). Utility of axial and radial diffusivity from diffusion tensor MRI as markers of neurodegeneration in amyotrophic lateral sclerosis. Brain Research, 1348, 156–164. 20513367 10.1016/j.brainres.2010.05.067

[brb3647-bib-0022] Moayedi, M. , Weissman‐Fogel, I. , Salomons, T. V. , Crawley, A. P. , Goldberg, M. B. , Freeman, B. V. , … Davis, K. D. (2012). White matter brain and trigeminal nerve abnormalities in temporomandibular disorder. Pain, 153, 1467–1477. 22647428 10.1016/j.pain.2012.04.003

[brb3647-bib-0023] Pasternak, O. , Kubicki, M. , & Shenton, M. E. (2015). In vivo imaging of neuroinflammation in schizophrenia. Schizophrenia Research, 173, 200–212. 26048294 10.1016/j.schres.2015.05.034 PMC4668243

[brb3647-bib-0024] Pasternak, O. , Westin, C.‐F. , Bouix, S. , Seidman, L. J. , Goldstein, J. M. , Woo, T.‐U. W. , … Kubicki, M. (2012). Excessive extracellular volume reveals a neurodegenerative pattern in schizophrenia onset. Journal of Neuroscience, 32, 17365–17372. 23197727 10.1523/JNEUROSCI.2904-12.2012 PMC3549332

[brb3647-bib-0025] Pleger, B. , Draganski, B. , Schwenkreis, P. , Lenz, M. , Nicolas, V. , Maier, C. , & Tegenthoff, M. (2014). Complex regional pain syndrome type I affects brain structure in prefrontal and motor cortex. PLoS One, 9, e85372. 24416397 10.1371/journal.pone.0085372 PMC3887056

[brb3647-bib-0026] Quarantelli, M. (2015). MRI/MRS in neuroinflammation: Methodology and applications. Clinical and Translational Imaging, 3, 475–489. 26705534 10.1007/s40336-015-0142-y PMC4679099

[brb3647-bib-0027] Rommel, O. , Malin, J.‐P. , Zenz, M. , & Jänig, W. (2001). Quantitative sensory testing, neurophysiological and psychological examination in patients with complex regional pain syndrome and hemisensory deficits. Pain, 93, 279–293. 11514087 10.1016/S0304-3959(01)00332-3

[brb3647-bib-0028] Roosendaal, S. D. , Geurts, J. J. G. , Vrenken, H. , Hulst, H. E. , Cover, K. S. , Castelijns, J. A. , … Barkhof, F. (2009). Regional DTI differences in multiple sclerosis patients. NeuroImage, 44, 1397–1403. 19027076 10.1016/j.neuroimage.2008.10.026

[brb3647-bib-0029] Schilder, J. C. M. , Schouten, A. C. , Perez, R. S. G. M. , Huygen, F. J. P. M. , Dahan, A. , Noldus, L. P. J. J. , … Marinus, J. (2012). Motor control in complex regional pain syndrome: A kinematic analysis. Pain, 153, 805–812. 22336720 10.1016/j.pain.2011.12.018

[brb3647-bib-0030] Scholz, J. , Klein, M. C. , Behrens, T. E. J. , & Johansen‐Berg, H. (2009). Training induces changes in white‐matter architecture. Nature Neuroscience, 12, 1370–1371. 19820707 10.1038/nn.2412 PMC2770457

[brb3647-bib-0031] Smith, S. M. (2002). Fast robust automated brain extraction. Human Brain Mapping, 17, 143–155. 12391568 10.1002/hbm.10062 PMC6871816

[brb3647-bib-0032] Smith, S. M. , Jenkinson, M. , Johansen‐Berg, H. , Rueckert, D. , Nichols, T. E. , Mackay, C. E. , … Behrens, T. E. J. (2006). Tract‐based spatial statistics: Voxelwise analysis of multi‐subject diffusion data. NeuroImage, 31, 1487–1505. 16624579 10.1016/j.neuroimage.2006.02.024

[brb3647-bib-0033] Smith, S. M. , Jenkinson, M. , Woolrich, M. W. , Beckmann, C. F. , Behrens, T. E. J. , Johansen‐Berg, H. , … Matthews, P. M. (2004). Advances in functional and structural MR image analysis and implementation as FSL. NeuroImage, 23(Suppl 1), S208–S219. 15501092 10.1016/j.neuroimage.2004.07.051

[brb3647-bib-0034] Smith, S. M. , & Nichols, T. E. (2009). Threshold‐free cluster enhancement: Addressing problems of smoothing, threshold dependence and localisation in cluster inference. NeuroImage, 44, 83–98. 18501637 10.1016/j.neuroimage.2008.03.061

[brb3647-bib-0035] Szabó, N. , Kincses, Z. T. , Párdutz, A. , Tajti, J. , Szok, D. , Tuka, B. , … Vécsei, L. (2012). White matter microstructural alterations in migraine: A diffusion‐weighted MRI study. Pain, 153, 651–656. 22244439 10.1016/j.pain.2011.11.029

[brb3647-bib-0036] Valkanova, V. , Eguia Rodriguez, R. , & Ebmeier, K. P. (2014). Mind over matter–what do we know about neuroplasticity in adults? International Psychogeriatrics, 26, 891–909. 24382194 10.1017/S1041610213002482

[brb3647-bib-0037] Van Hecke, W. , Leemans, A. , D'Agostino, E. , De Backer, S. , Vandervliet, E. , Parizel, P. M. , & Sijbers, J. (2007). Nonrigid coregistration of diffusion tensor images using a viscous fluid model and mutual information. IEEE Transactions on Medical Imaging, 26, 1598–1612. 18041274 10.1109/TMI.2007.906786

[brb3647-bib-0038] van Velzen, G. A. J. , Rombouts, S. A. R. B. , van Buchem, M. A. , Marinus, J. , & van Hilten, J. J. (2016). Is the brain of complex regional pain syndrome patients truly different? European Journal of Pain, 20, 1622–1633. 27161331 10.1002/ejp.882

[brb3647-bib-0039] Wang, Y. , Gupta, A. , Liu, Z. , Zhang, H. , Escolar, M. L. , Gilmore, J. H. , … Styner, M. (2011). DTI registration in atlas based fiber analysis of infantile Krabbe disease. NeuroImage, 55, 1577–1586. 21256236 10.1016/j.neuroimage.2011.01.038 PMC3062693

[brb3647-bib-0040] Yu, D. , Yuan, K. , Qin, W. , Zhao, L. , Dong, M. , Liu, P. , … Tian, J. (2013). Axonal loss of white matter in migraine without aura: A tract‐based spatial statistics study. Cephalalgia, 33, 34–42. 23150889 10.1177/0333102412466964

[brb3647-bib-0041] Zhang, H. , Avants, B. B. , Yushkevich, P. A. , Woo, J. H. , McCluskey, L. F. , Elman, L. B. , … Gee, J. C. (2007). High‐dimensional spatial normalization of diffusion tensor images improves the detection of white matter differences: An example study using amyotrophic lateral sclerosis. IEEE Transactions on Medical Imaging, 26, 1585–1597. 18041273 10.1109/TMI.2007.906784

[brb3647-bib-0042] Zhang, S. , Peng, H. , Dawe, R. J. , & Arfanakis, K. (2011). Enhanced ICBM diffusion tensor template of the human brain. NeuroImage, 54, 974–984. 20851772 10.1016/j.neuroimage.2010.09.008 PMC2997145

[brb3647-bib-0043] Zhang, H. , Yushkevich, P. A. , Rueckert, D. , & Gee, J. C. (2010). A computational white matter atlas for aging with surface‐based representation of fasciculi In Fischer B., Dawant B. M., & Lorenz C. (Eds.), Biomedical image registration (pp. 83–90). Berlin, Heidelberg, Germany: Springer.

[brb3647-bib-0044] Zhou, G. , Hotta, J. , Lehtinen, M. K. , Forss, N. , & Hari, R. (2015). Enlargement of choroid plexus in complex regional pain syndrome. Scientific Reports, 5, 14329. 26388497 10.1038/srep14329 PMC4585686

