# Supplementary material for: Complex regional pain syndrome: The matter of white matter?
Source: Brain Behav. 2017 Apr 5;7(5):e00647. doi: 10.1002/brb3.647 (PMC5434177; doi:10.1002/brb3.647)
Supplement: Supplementary file 1 [file BRB3-7-e00647-s001.docx]

Supplementary Table SI

The mean ± SD active ranges of motion (AROM) and strength in the healthy and affected hand of the 12 CRPS type 1 patients. The AROMs were mainly measured with goniometer (angle), but for finger flexions the distance (cm) from the tip of the finger to the palm of the hand in maximum finger flexion, and for the thumb-opposition the distance from the tip of the thumb to the tip of the finger 5 in maximum opposition were applied. Grip strength was measured with Jamar dynamometer (position II), whereas the strengths for tip and lateral pinch were measured with a B&L Engineering pinch gauge.

|  | Healthy hand | Affected hand | p (uncorrected) |
| --- | --- | --- | --- |
| AROM |  |  |  |
| Fingers 2–5 flexion [cm]^1^ | 0.0 ± 0.0 | 0.6 ± 1.1 | 0.110 |
| Fingers 2–5 extension [angle]^1^ | 0.0 ± 0.0 | 1.7 ± 3.7 | 0.151 |
| Thumb opposition [cm] | 0.0 ± 0.0 | 0.3 ± 1.0 | 0.343 |
| Thumb radial abduction [angle] | 75.0 ± 11.7 | 55.4 ± 12.7 | 0.0005* |
| Wrist volar flexion [angle] | 81.2 ± 5.7 | 64.4 ± 17.2 | 0.006* |
| Wrist dorsal flexion [angle] | 76.8 ± 7.9 | 56.5 ± 19.0 | 0.00002* |
| Elbow flexion [angle] | 150.0 ± 0.0 | 150.0 ± 0.0 | - |
| Elbow extension [angle] | 0.0 ± 0.0 | 0.0 ± 0.0 | - |
| Shoulder flexion [angle] | 170.0 ± 9.0 | 144.2 ± 32.8 | 0.013* |
| Strength [kg] |  |  |  |
| Tip pinch | 4.1 ± 0.9 | 3.0 ± 1.4 | 0.019* |
| Lateral pinch | 6.7 ± 1.7 | 5.3 ± 2.8 | 0.057 |
| Grip strength | 24.2 ± 10.5 | 13.9 ± 10.9 | 0.016* |

* Statistically significant impairment in the affected hand compared with the healthy hand (two-tailed paired *t* test).

^1^ The average of AROMs of fingers 2–5
