# Supplementary material for: Complex regional pain syndrome: The matter of white matter?
Source: Brain Behav. 2017 Apr 5;7(5):e00647. doi: 10.1002/brb3.647 (PMC5434177; doi:10.1002/brb3.647)
Supplement: Supplementary file 2 [file BRB3-7-e00647-s002.pdf]

Supplementary material

Motor symptom severity index (MSSI)

This study was a part of a larger set of studies addressing the central manifestations of upper-limb CRPS, in which we assessed the patients' motor symptoms and signs in five aspects: (1) movement pain and (2) upper-limb disability were assessed with questionnaires, whereas (3) hand dexterity, (4) joint movability, and (5) muscle strengths were measured in physical examination performed by a skilled physiotherapist.

For the movement pain, patients were asked to rate their maximum movement-related pain during the previous week on an 11-point numeric rating scale (NRS-11; 0 = no pain, 10 = extreme pain). The upper-limb disability was characterized using Disabilities of Arm, Shoulder, and Hand questionnaire (DASH, Institute for Work & Health; http://www.dash.iwh.on.ca/home; [Hudak et al., 1996]). In the physical examinations, the hand dexterity was assessed with a nine-hole peg test (9-HPT), and the joint movability by measuring the active range of motion (AROM) in the flexion and extension of fingers 2–5, wrist, and elbow, the opposition and radial abduction of the thumb, and the flexion of the shoulder. The muscle strengths were measured for hand grip, tip-pinch and lateral-pinch.

With the abundance of motor measures, we wanted to know how the overall motor-symptom severity associated to DTI measures. To this end, we created a compound score called motor symptom severity index (MSSI). In order to weigh different motor symptoms equally in the MSSI, we included only one measure from each of the five motor aspects assessed. For the movement pain (NRS-11, mean ± SD 7.6 ± 1.7), upper-limb disability (DASH, 56.8 ± 16.6), and hand dexterity (9-HPT, 20.8 ± 5.5 s) there was only one measure each, so no selection was required. For the joint movability and muscle strengths we had the possibility to select the most suitable one from multiple measures. The total wrist AROM (sum of extension and flexion AROM, 120.9 ± 34.9°) was included, because the wrist AROMs (1) were sensitive to effects of CRPS, which means that the affected-hand measures were statistically different from those of the healthy hand (see

Supplementary Table SI), (2) were easy and unambiguous to measure, and (3) reflected the distal functions of the upper limb which CRPS typically affects. For the same reasons, both the tip pinch and grip strength could have been included, but the grip strength (13.9 ± 10.9 kg) was selected because of its more common usage in clinical practice.

The MSSI was calculated by summing together the z-scores of its subcomponents; before the sum, the AROM and grip strength were multiplied by −1, so that for all the measures a greater score indicate more severe symptoms.

According to the Shapiro-Wilk test, the MSSI was normally distributed. The MSSI correlated statistically significantly with four-out-of-five of its subcomponents: DASH (Pearson's $r = 0.86$, $p < 0.001$), 9-HPT ($r = 0.83$, $p < 0.001$), total-wrist AROM ($r = -0.63$, $p < 0.05$), and grip strength ($r = -0.78$, $p < 0.005$). The correlation with movement pain ($r = 0.52$, $p = 0.085$) did not reach statistical significance. Figure 1S shows the MSSI values as a function of its subcomponents.

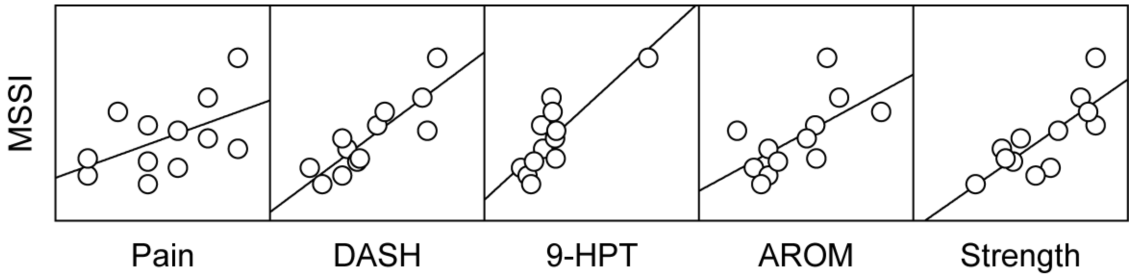

Figure 1S, MSSI values of each subject plotted as a function of movement pain (NRS-11), DASH, nine-hole peg test (9-HPT), active range of wrist motion (AROM), and grip strength (Strength). The scales for AROM and Strength are reversed for visualization purposes. The black line represents the linear fit.
